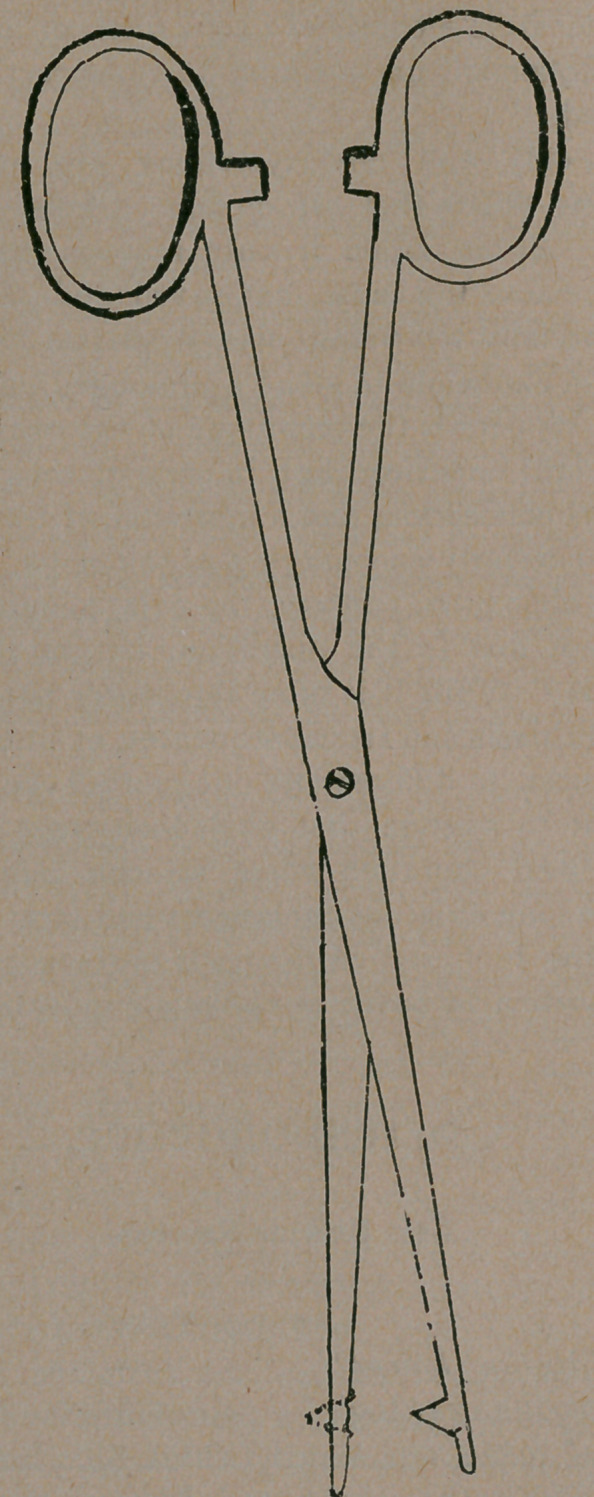# New Uterine Forceps

**Published:** 1895-11

**Authors:** Thomas J. Turpin

**Affiliations:** Laredo, Texas


### Correspondence.

### New Uterine Forceps.

LAREDO, TEXAS, October 3, 1895.

*Editor Texas Medical Journal:*

I enclose a rough drawing of a pair of uterine forceps which I have lately had made, and which I think fill a long felt want.

In operation upon the cervix uteri, I have often found that the ordinary volcellum forceps had to be reapplied two or three times, thus making half a dozen or more holes in the uterine tissue. In order to avoid this, I have been in the habit of passing a stout silk ligature through both lips of the cervix, and have found that these answered better than the volcellun to draw down the os.

The chief objection to this is, however, that one is liable to cut the ligature just at the time when it is most necessary to make traction in order to bring the womb down to the vulva.

My new forceps does away with the above mentioned difficulties. The female blade being introduced into the cervical canal, the male blade, with the pin, is then clamped, pushing the pin through the uterine tissue until the pin fills up the hole in the female blade.

Another pair of forceps can then be applied in the same way to the other side of the cervix, and the uterus drawn down to the vulva.

The female blade, when properly applied, fits into the cervix and covers just the proper breadth of mucous membrane that should be left in trachelorrhaphies involving both sides of the cervix.

I have found these forceps so convenient in operation upon the uterus that I send you this for publication in your valuable journal, in order that the profession may profit by it.

Very truly,

THOS. J. TURPIN, M. D.

## Figures and Tables

**Figure f1:**